# Stereotactic Body Radiation Therapy (SBRT) for a Patient with a Myocardial Metastasis: A Case Report

**DOI:** 10.3390/curroncol28010041

**Published:** 2021-01-12

**Authors:** Aneesh Dhar, Elysia Donovan, Darryl Leong, Sebastien J. Hotte, Anand Swaminath

**Affiliations:** 1 Juravinski Cancer Centre, Hamilton, ON L8V 5C2, Canada; aneesh.dhar@medportal.ca (A.D.); hotte@hhsc.ca (S.J.H.); 2 Sunnybrook Health Sciences Centre, Toronto, ON M4N 3M5, Canada; elysia.donovan@sunnybrook.ca; 3 The Population Health Research Institute, McMaster University& Hamilton Health Sciences, Hamilton, ON L8L 2X2, Canada; leongd@phri.ca; 4 Department of Oncology, McMaster University, Hamilton, ON L8S 4L8, Canada

**Keywords:** stereotactic body radiation therapy (SBRT), myocardial metastasis, cardiac magnetic resonance imaging

## Abstract

Metastatic lesions of the heart are rare but have the potential to cause significant morbidity. We describe the case of a patient with renal cell carcinoma who presented with shortness of breath and palpitations and was found to have a metastatic myocardial lesion causing arrythmia. He received stereotactic body radiation therapy (SBRT) to alleviate symptoms and provide local control. SBRT planning was executed using a four-dimensional computed tomography (4DCT) scan to account for respiratory and cardiac motion. Images from a planning magnetic resonance imaging (MRI) scan and a gated diagnostic MRI scan of the heart were fused with the 4DCT to assist with delineating the tumour. A dose of 30 Gy in five fractions was delivered without incident. The patient’s cardiac MRI at two months post-treatment showed stability of his cardiac lesion. He subsequently died of distant disease progression, without any recurrence of his cardiac symptoms. SBRT may be considered for patients who present with a symptomatic metastatic cardiac lesion.

## 1. Introduction

While pericardial and mediastinal metastases adjacent to the heart are a relatively common occurrence in patients with an advanced malignancy, metastasis within the myocardium are rare [1]. Treatment of cardiac metastases depends on the severity of symptoms and the acuity of presentation. Radiation therapy can alleviate symptoms in this setting. With advances in radiation oncology, more precise treatments can be delivered to patients with myocardial metastases [2,3,4,5,6]. Stereotactic body radiation therapy (SBRT) is a highly precise ablative radiation treatment with excellent local control rates and favourable toxicity profiles for systemic metastases [7,8,9]. However, there is a paucity of studies on the use of SBRT for myocardial metastases. In this report, we describe the case of a patient treated with SBRT for a symptomatic myocardial metastasis.

## 2. Case Description

We describe a 54-year-old man with renal cell carcinoma (RCC) diagnosed nine years earlier. His RCC was initially treated with laparoscopic nephrectomy (pT2, cN0 by the 7th edition American Joint Committee on Cancer Staging Manual). The patient was surveilled, and a pancreatic metastasis was identified three years later. The patient was treated with sunitinib for two years and then developed intolerable neurologic side effects and stopped the medication. A few months later, his pancreatic lesion progressed, and a new adrenal metastasis developed. These were treated with SBRT and were locally controlled for over 12 months. He continued to not take sunitinib and developed new metastases in the liver and lungs two years after receiving SBRT to the pancreas and adrenal gland. The patient then decided to retry sunitinib but had progression of his disease shortly thereafter. He was then switched to nivolumab for nine months, with a partial response.

**Figure 1:**
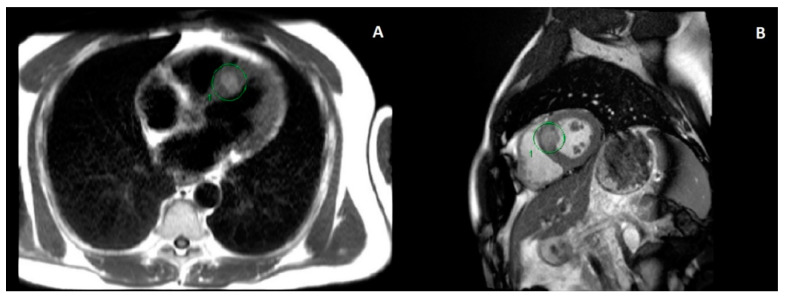
Magnetic resonance imaging (MRI) of the thorax and upper abdomen using gadolinium contrast and cardiac gating. Images from left to right show axial (**A**) and sagittal (**B**) views of the metastatic myocardial septum lesion (circled) prior to any treatment.

This patient then presented with a one-week history of shortness of breath, palpitations and presyncopal symptoms. A physical examination revealed an irregularly irregular rhythm on auscultation. An electrocardiogram (ECG) showed multiple ventricular ectopic beats. The patient was sent to the emergency department to have an urgent cardiology consultation to rule out an immunotherapy-mediated myocarditis. He was admitted in hospital and was monitored with telemetry. Bloodwork and serum troponin level were normal. Cardiac magnetic resonance imaging (MRI) showed a 2.8-cm metastatic lesion within the myocardial septum (Figure 1). In retrospect, this was noted on CT imaging 18 months and a few weeks prior to the cardiac MRI, where the lesion measured 1.3 cm and 2.4 cm, respectively. Ultimately, this lesion was felt to be the aetiology of his symptoms.

**Figure 2:**
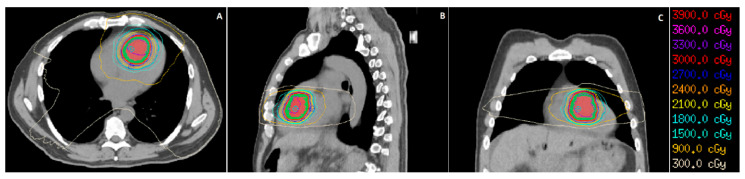
Isodose distribution for this patient’s treatment, shown with representative images in the axial (**A**), sagittal (**B**), and coronal (**C**) planes of the patient’s CT simulation scan. The gross tumour volume (GTV) and planning target volume (PTV) are shown as colour-washed areas in red and green, respectively. Isodose lines of varying doses and colours are shown as well.

The cardiology team started the patient on beta blocker therapy, which relieved his symptoms. He was subsequently discharged from hospital with nivolumab held, as imaging done prior to his admission showed systemic disease progression. He was assessed for SBRT to his cardiac lesion with the goal of local control and symptom relief. The patient received SBRT 30 Gy in five fractions and was treated every other day. Simulation scans for SBRT were done with a four-dimensional computed tomography (4DCT) scan to account for respiratory and cardiac motion and with triphasic contrast-enhanced MRI to delineate the tumour location. The patient’s cardiac-gated diagnostic MRI was fused with the 4DCT and MRI simulation scans to further help localise the lesion. The patient was immobilised with a vacuum cushion (Vac-Lok^TM^, CIVCO Radiotherapy, Coralville, IA, USA) and was placed supine during simulation and treatment, with both arms raised above his head. Prior to each treatment, the patient had a kilovoltage cone beam CT (CBCT) scan to verify the position of the heart compared to the 4DCT simulation scan. A volumetric modulated arc therapy (VMAT) technique was used to deliver the treatment using 6-MV photons. The prescription dose (30 Gy) was required to cover at least 95% of the planning target volume (PTV). Organs at risk were delineated, and dose constraints (Table 1) were derived from a study of SBRT treatments for central non-small cell lung cancer (NSCLC) lesions [10]. The isodose distribution of the patient’s treatment plan is shown (Figure 2).

**Figure 3:**
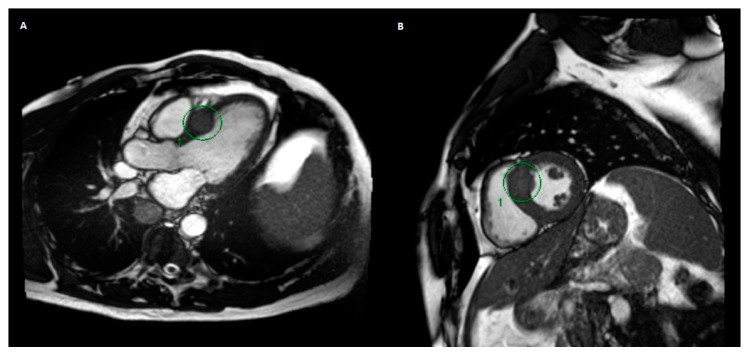
MRI of the thorax and upper abdomen done three months after radiation treatment, showing stability of the metastatic myocardial lesion (circled). No gadolinium contrast was used for this scan, as the patient had an acute kidney injury at the time. Representative axial (**A**) and sagittal (**B**) images are shown on the left and right, respectively.

Overall, the patient tolerated his treatment course well. During his treatment, he had one episode of chest pain that self-resolved without physician intervention (grade 1 cardiac pain, by Common Terminology Criteria for Adverse Events (CTCAE) v5.0), as well as some mild dyspnoea on exertion (grade 1 dyspnoea by CTCAE v5.0). Two weeks later, he had an ECG showing no significant abnormalities. Six weeks after treatment, an echocardiogram showed a left ventricular ejection fraction (LVEF) of 63% and no visualised myocardial lesion. An echocardiogram done five years prior showed a LVEF of 53%, and no myocardial lesion was seen. An MRI performed two months after treatment (Figure 3) showed stability of his cardiac lesion, although no contrast was given for his MRI scan due to concerns of renal dysfunction at the time.

Ten months after SBRT treatment, the patient developed new metastatic lesions in the lungs and brain, which were treated with radiation. The patient ultimately had progressive neurologic symptoms and was transferred to hospice, where he died soon after. He did not experience any further recurrence of his chest pain or dyspnoea. His last available CT and MRI scans occurred eight months and two months after his treatment, both of which showed stability of his cardiac lesion.

This study was conducted according to the guidelines of the Declaration of Helsinki. Informed consent was obtained from the patient, as per our institution’s Ethics Review Board.

## 3. Discussion

We present the rare case of a patient with a metastatic cardiac lesion who received SBRT to provide local control and alleviate symptoms. This was in the context of systemic disease in the lungs, liver, and brain. He had disease progression on sunitinib and nivolumab, and other systemic therapy options could not be considered until his cardiac function stabilised. Similarly, surgical options were not an option. Therefore, SBRT provided an opportunity to provide symptomatic control while sparing the adjacent heart.

There were many challenges with SBRT delivery in this case. Firstly, the safety of high-dose radiotherapy to the heart has not been established. This is, in part, due to the paucity of prospective data in the use of SBRT for cardiac lesions. The Radiation Therapy Oncology Group(RTOG) 0813 trial investigated the use of SBRT for centrally located NSCLC lesions [10]. In this trial, the maximum tolerated dose was defined as the maximum dose a patient could receive such that the probability of grade 3+ toxicity was less than 20%. The maximum tolerated dose was 60 Gy in five fractions, with an associated grade 3+ toxicity rate (CTCAE v4.0) of 7.2%, which included cardiac and noncardiac toxicities. Overall, one hundred patients received SBRT at various dose levels (50–60 Gy in five fractions) in this trial, of which there were two patients and one patient with grade 3 and grade 5 cardiac toxicity, respectively. This suggests a grade 3+ cardiac toxicity rate of approximately 3% with this treatment. The constraints used in this trial were used for our patient’s case, as it was thought that this trial would accurately represent the risk of adverse events in organs at risk in the thorax and mediastinum when using SBRT.

There were two Italian case series where patients received SBRT for cardiac primary and secondary malignancies [5,6]. The patients in the first case series (*n* = 3) received either 24 Gy in three fractions or 30 Gy in five fractions and had stability or partial response of their disease on a cardiac MRI at six months after SBRT. The patients in the second case series (*n* = 16) received either 36 Gy in three fractions or 30 Gy in three fractions. After SBRT, 12 of 16 (75%) patients had a local response or stability of disease. The median time to local failure was 5.2 months. Four patients died, all from distant progression of the disease.

In the first Italian study, there were no changes on the echocardiography or ECG and no rise in cardiac enzymes for all three patients after SBRT. The patients did not describe any concerning symptoms. In the second case series, all patients denied cardiac symptoms, and all had no changes on their ECGs. One patient developed an asymptomatic pericardial effusion, which self-resolved on subsequent imaging. Another patient developed grade 2 esophagitis (CTCAE version 4.0), which required anti-inflammatory medications for treatment.

In our case, the patient did have mild chest pain and dyspnoea on exertion, although these self-resolved without intervention. There was improvement of the ECG findings after beta blocker therapy was started in hospital, and this continued for the remainder of the patient’s life. The troponin level prior to SBRT was normal and was not repeated after SBRT. An echocardiogram after SBRT did not reveal the lesion. His cardiac-gated MRI done prior to and after SBRT showed stability of the lesion. CT imaging of the chest was also done before and after SBRT, but these did not show the lesion as clearly as the cardiac-gated MRI.

Another challenge in this case was the delineation of the tumour for SBRT planning. Cardiac gating was not possible on the MRI simulation scans. The assessment of cardiac muscular motion through the cardiac cycle was difficult on both CT and MRI. The cardiac-gated diagnostic MRI was helpful in assisting with tumour delineation; however, the lesion was contoured generously to ensure the treatment encompassed it. Nevertheless, we recommend utilising cardiac-gated MRI, if available, to assist with delineating cardiac lesions during SBRT planning.

## 4. Conclusions

In summary, we described a case of a patient receiving palliative radiation therapy to the heart for a metastatic lesion using SBRT. Our case showed that this is feasible and well-tolerated in the short term, with few side effects during treatment. At the last available imaging, the patient had stability of his cardiac lesion. Cardiac-gated MRI was the best imaging modality to visualise this lesion and was useful for delineating the lesion for treatment planning. Long-term follow-up of patients receiving radiation to the heart in prospective trials are needed to help clarify the safety of this technique moving forward.

## Figures and Tables

**Table 1:** Stereotactic body radiation therapy (SBRT) treatment planning dose statistics.

Region of Interest	Dose Volume Histogram Parameter	Constraint as per RTOG 0813	Value Achieved for This Case
PTV	R100	<1.2	1.04
	R50	<4.0	3.68
	D2cm	<62.0%	51.6%
Spinal Cord	D (0.0 mL)	<30 Gy	1.59 Gy
	D (0.25 mL)	<22.5 Gy	1.47 Gy
	D (0.5 mL)	<13.5 Gy	1.43 Gy
Skin	D (0.0 mL)	<32 Gy	9.40 Gy
	D (10.0 mL)	<30 Gy	6.38 Gy
Bilateral Lung	V (20 Gy)	<10%	<1%
	D (1500 mL)	<12.5 Gy	2.09 Gy
	D (1000 mL)	<13.5 Gy	3.13 Gy
Esophagus (nonadjacent to PTV)	D (0.0 mL)	<105% of prescription dose	14.7%
	D (5.0 mL)	27.5	3.42 Gy
Heart and Pericardium	D (0.0 mL)	<105% of prescription dose	113.7% *
	D (15.0 mL)	32	31.82 Gy
Great Vessels (nonadjacent to PTV)	D (0.0 mL)	<105% of prescription dose	12.0%
	D (10.0 mL)	47	2.70 Gy
Trachea (nonadjacent to PTV)	D (0.0 mL)	<105% of prescription dose	0.7%
	D (4.0 mL)	18	0.16 Gy
Proximal Bronchial Tree (nonadjacent to PTV)	D (0.0 mL)	<105% of prescription dose	16.7%
	D (4.0 mL)	18	3.73 Gy

A list of regions of interest, organs at risk, plan parameters, constraints from the Radiation Therapy Oncology Group (RTOG) 0813 study, and corresponding values achieved for this patient’s treatment plan. An asterisk (*) represents a plan parameter for which the dose constraint from RTOG 0813 was not achieved in this patient’s treatment plan. This occurred due to the planning target volume (PTV) being located within the organ at a risk contour of the heart. The R100 represents the ratio of the prescription isodose volume to the volume of the PTV. The R50 represents the ratio of the 50% isodose volume to the volume of the PTV. The D2cm represents the maximum dose, as a percentage of the prescription dose, that is 2 cm away from the PTV. D (X mL) represents the minimum dose, in Gy, found in the hottest region of the volume, equal to X millilitres. V (Y Gy) represents the volume, as a percentage of the total volume of the region of interest, that has received at least Y Gy dose.

## Data Availability

Data sharing not applicable.
